# Rituximab Outcomes and Predictors of Treatment Failure in Primary Membranous Nephropathy: A Retrospective Cohort Study

**DOI:** 10.3390/jcm15187067

**Published:** 2026-09-11

**Authors:** Saide Elif Güllülü Boz, Fazıl Çağrı Hunutlu, Esra Çelik, Abdullah İbrahim Çalışır, Halime Soyak Kabaca, Cuma Bülent Gül, Abdülmecit Yıldız, Alparslan Ersoy, Mustafa Güllülü, Ayşegül Oruç

**Affiliations:** 1 Department of Nephrology, Faculty of Medicine, Bursa Uludag University, 16059 Bursa, Türkiye; 2 Department of Hematology, Bursa City Training and Research Hospital, 16110 Bursa, Türkiye; 3 Department of Internal Medicine, Avcilar Murat Koluk State Hospital, 34320 Istanbul, Türkiye

**Keywords:** anti-PLA2R antibody, clinical remission, membranous nephropathy, rituximab, treatment failure

## Abstract

**Background/Objectives:** Rituximab (RTX) is an established treatment for primary membranous nephropathy (PMN); however, real-world data on predictors of response and the influence of previous treatment status remain limited. We evaluated outcomes and predictors of treatment failure in PMN patients treated with RTX. **Methods:** This retrospective cohort study included 58 adults with biopsy-proven PMN who received RTX between 2013 and 2023. Clinical remission at 6 and 9 months, treatment failure (persistent non-response or relapse after remission), and failure-free survival were evaluated. Outcomes were compared according to baseline anti-phospholipase A2 receptor (anti-PLA2R) antibody levels and previous treatment status. **Results:** Clinical remission increased significantly from 60.3% at 6 months to 72.4% (42/58) at 9 months (*p* < 0.001). Patients with baseline anti-PLA2R levels ≤ 150 RU/mL achieved significantly higher remission rates at both time points. The pattern of treatment failure also differed, with persistent refractory disease predominating at higher antibody levels and relapse after remission at lower levels. Baseline serum albumin was the only variable independently associated with treatment failure (odds ratio 0.18, 95% confidence interval 0.04–0.61; *p* = 0.010). Outcomes were comparable among treatment-naive, relapsing, and treatment-refractory patients at the predefined 9-month assessment. Infections occurred in 7 patients (12.1%). **Conclusions:** RTX achieved favorable clinical outcomes in PMN, with remission continuing to improve beyond 6 months. Baseline anti-PLA2R antibody levels and serum albumin provided complementary prognostic information. Previous treatment status was not associated with remission or treatment failure at the predefined 9-month assessment and was not independently associated with failure-free survival after adjustment for baseline serum albumin, although this finding should be interpreted cautiously given the limited sample size and unequal follow-up.

## 1. Introduction

Primary membranous nephropathy (PMN) is one of the leading causes of nephrotic syndrome in adults. It is characterized by subepithelial immune complex deposition along the glomerular basement membrane, resulting in nephrotic-range proteinuria, hypoalbuminemia, and an increased risk of progressive kidney dysfunction. The clinical course of PMN is highly heterogeneous. While spontaneous remission may occur in some patients, others experience persistent nephrotic syndrome or progressive kidney function decline necessitating immunosuppressive therapy [1].

The management of PMN has evolved considerably over the past decade with advances in the understanding of its immunopathogenesis [2]. B-cell depletion with rituximab (RTX) has emerged as an effective therapeutic strategy, supported by several randomized controlled trials. The GEMRITUX trial first demonstrated that although RTX did not significantly improve remission rates at 6 months, extended follow-up revealed significantly higher remission rates than supportive therapy alone [3]. Subsequently, the MENTOR trial established RTX as non-inferior to cyclosporine at 12 months and superior in maintaining remission at 24 months [4]. More recently, the STARMEN and RI-CYCLO trials further compared RTX-containing regimens with cyclophosphamide-based treatment strategies [5,6]. Taken together, these landmark trials established RTX as a cornerstone in the management of PMN and formed the evidence base for the 2021 KDIGO Clinical Practice Guideline [1].

Despite these advances, several clinically important questions remain unresolved. Although randomized controlled trials have demonstrated the efficacy of RTX, they enrolled relatively selected patient populations under standardized treatment protocols. In routine clinical practice, treatment decisions are often individualized according to disease severity, previous immunosuppressive exposure, and physician preference. A recent real-world study suggested that previous treatment status may also influence RTX response, with treatment-refractory patients achieving lower remission rates than treatment-naive or relapsing patients [7]. However, these findings require validation in independent cohorts. Moreover, predictors of RTX response remain incompletely understood, particularly in heterogeneous real-world cohorts. The prognostic value of baseline anti-phospholipase A2 receptor (anti-PLA2R) antibody levels and other clinical factors for predicting long-term treatment failure also remains uncertain.

Therefore, we conducted a retrospective cohort study to evaluate the clinical outcomes of RTX in patients with PMN. Our primary objective was to identify baseline predictors of long-term treatment failure. Secondary objectives were to assess clinical remission at 6 and 9 months, to describe longitudinal changes in proteinuria and serum albumin, and to compare outcomes according to baseline anti-PLA2R antibody levels and previous treatment status.

## 2. Materials and Methods

### 2.1. Study Design and Patient Population

This retrospective cohort study included adult patients (≥18 years) with biopsy-proven PMN who received RTX at the Department of Nephrology, Faculty of Medicine, Bursa Uludag University, between January 2013 and December 2023. A total of 72 patients were screened for eligibility. Eight patients with secondary membranous nephropathy, four with insufficient follow-up, and two with incomplete baseline data, including an unavailable baseline anti-PLA2R antibody measurement, were excluded. The final study cohort comprised 58 patients, all of whom had a baseline anti-PLA2R antibody measurement available.

### 2.2. Data Collection

Demographic, clinical, laboratory, and treatment-related data were retrieved from electronic medical records. Baseline variables included age, sex, serum albumin, 24 h urinary protein excretion, serum creatinine, estimated glomerular filtration rate (eGFR), serum anti-PLA2R antibody levels, KDIGO risk category, and previous immunosuppressive treatment history.

Baseline serum anti-PLA2R antibody measurement was available for all patients and was a prerequisite for study inclusion. Follow-up anti-PLA2R antibody testing, however, was performed at external laboratories on a self-paid basis and was therefore not routinely available. Consequently, serial changes in anti-PLA2R antibody levels and immunological remission could not be evaluated as treatment outcomes, and all anti-PLA2R analyses in this study are based on baseline measurements. The eGFR was calculated for all patients using the 2021 Chronic Kidney Disease Epidemiology Collaboration (CKD-EPI) creatinine equation, applied uniformly to all patients irrespective of the year of enrollment.

### 2.3. Rituximab Treatment

All patients received RTX according to the institutional treatment protocol. RTX was administered intravenously at a dose of 1 g on days 1 and 15. Retreatment with RTX was performed at the discretion of the treating physician based on disease activity, clinical remission status, anti-PLA2R antibody levels (when available), and overall clinical assessment. Depending on disease severity, previous treatment history, and the treating physician’s clinical judgment, RTX was administered either as monotherapy or in combination with corticosteroids and/or CNIs.

All patients received an angiotensin-converting enzyme inhibitor (ACEi) or an angiotensin receptor blocker (ARB). Lipid-lowering agents and anticoagulant therapy were prescribed according to individual clinical indications. Sodium-glucose cotransporter-2 inhibitors (SGLT2i) were not routinely prescribed because reimbursement restrictions limited their standard use during the study period.

### 2.4. Outcomes and Definitions

Patients were categorized according to their treatment history before RTX as treatment-naive (no previous immunosuppressive therapy), relapsing (recurrence of nephrotic syndrome after a previous complete or partial remission following immunosuppressive therapy), and treatment-refractory (persistent nephrotic syndrome despite previous immunosuppressive therapy).

Patients were also stratified according to the risk of progressive loss of kidney function as defined by the 2021 KDIGO Clinical Practice Guideline [1]. Moderate risk was defined as a normal eGFR with proteinuria > 3.5 g/day that did not decrease by more than 50% with conservative therapy, in the absence of high-risk criteria. High risk was defined as an eGFR < 60 mL/min/1.73 m^2^ and/or proteinuria > 8 g/day persisting for more than 6 months, or a normal eGFR with proteinuria > 3.5 g/day and no decrease of more than 50% with conservative therapy together with at least one additional marker of high immunological or clinical risk, such as a serum albumin < 2.5 g/dL or an anti-PLA2R antibody level > 50 RU/mL. Very high risk was defined as life-threatening nephrotic syndrome or rapid, otherwise unexplained deterioration of kidney function. Urinary low-molecular-weight proteins and the selectivity index were not measured routinely in clinical practice and were therefore not used for risk assignment. No patient in the present cohort met the criteria for the low-risk category, consistent with the indication for immunosuppressive therapy in all included patients. The anti-PLA2R threshold of 50 RU/mL was used only for KDIGO risk assignment, as specified in the guideline, whereas the separate threshold of 150 RU/mL used for the outcome comparisons throughout this study was adopted from previous reports and was not derived from the present data.

The primary outcome was treatment failure during follow-up, defined as persistent non-response at 9 months after RTX administration or relapse after achieving complete or partial remission. Because the clinical response to RTX may be delayed, consistent with previous studies, persistent non-response was assessed at 9 months rather than at the conventional 6-month time point. Secondary outcomes included clinical remission at 6 and 9 months, failure-free survival, and comparisons of outcomes according to baseline anti-PLA2R antibody levels and previous treatment status.

For failure-free survival analysis, the event was defined as persistent non-response at the 9-month assessment or relapse after achieving remission, whichever occurred first. Patients without treatment failure were censored at their last follow-up visit. For patients with persistent non-response, the event was recorded at the 9-month outcome assessment, using the follow-up visit closest to 9 months after RTX initiation (observed range 8.3–10.9 months); no event was recorded before this assessment. For patients who relapsed after achieving remission, the event time corresponded to the date of relapse identified during follow-up.

Clinical remission was defined according to the 2021 KDIGO Clinical Practice Guideline [1]. Complete remission (CR) was defined as proteinuria < 0.3 g/day with stable kidney function. Partial remission (PR) was defined as proteinuria < 3.5 g/day together with at least a 50% reduction from baseline and stable kidney function. Clinical remission comprised both complete and partial remission.

### 2.5. Statistical Analysis

Continuous variables were tested for normality using the Shapiro–Wilk test and are presented as median (interquartile range, IQR), while categorical variables are presented as number (%). Given the non-normal distribution of most variables and the limited sample size, non-parametric methods were used throughout. Baseline characteristics were compared among the treatment-naive, relapsing, and treatment-refractory groups using the Kruskal–Wallis test for continuous variables and Fisher’s exact test for categorical variables; for two-group comparisons, the Mann–Whitney U test was used in place of the Kruskal–Wallis test.

Within-patient changes in serum albumin and proteinuria from baseline to months 6 and 9 were analyzed using the Friedman test, followed by pairwise Wilcoxon signed-rank tests for baseline-versus-month-6, baseline-versus-month-9, and month-6-versus-month-9 comparisons. Changes in categorical treatment response between the 6- and 9-month assessments were analyzed using the Wilcoxon signed-rank test.

Time to treatment failure, defined as the interval from RTX initiation to relapse, refractoriness, or last follow-up, was estimated using the Kaplan–Meier method and compared among the three treatment-history groups with the log-rank test.

To evaluate whether the observed difference in failure-free survival across treatment-history groups persisted after adjustment for baseline serum albumin, which was the strongest predictor identified in the logistic regression analysis, an exploratory Cox proportional hazards model was fitted. The proportional hazards assumption was assessed using Schoenfeld residuals.

Candidate predictors of treatment failure—age, sex, baseline albumin, baseline proteinuria (<8000 mg/day vs. ≥8000 mg/day), baseline creatinine, baseline eGFR, anti-PLA2R titer > 150 RU/mL, very-high clinical risk category versus others, and refractory disease at baseline—were first screened in univariable logistic regression models. Variables associated with treatment failure at *p* < 0.20 were entered into a multivariable logistic regression model; the KDIGO risk category was additionally retained a priori because of its established clinical relevance. Odds ratios (ORs) with 95% confidence intervals (CIs) are reported.

Given the limited number of treatment-failure events relative to the number of candidate predictors, the number of events per variable (EPV) was calculated for the final multivariable model, and internal validation was performed using bootstrap resampling (1000 resamples, sampling with replacement) to estimate the optimism-corrected C-statistic according to Harrell’s method.

All statistical tests were two-tailed, and a *p*-value < 0.05 was considered statistically significant. Analyses were performed in R (version 4.5.2; R Foundation for Statistical Computing, Vienna, Austria) using the readxl, dplyr, ggplot2, survival, and survminer packages.

## 3. Results

### 3.1. Baseline Characteristics

A total of 58 patients with biopsy-proven PMN who received RTX were included in the study. According to previous treatment status, 13 (22.4%) patients were treatment-naive, 22 (37.9%) had relapsing disease, and 23 (39.7%) had treatment-refractory disease. Baseline demographic and clinical characteristics are summarized in Table 1.

Baseline demographic and clinical characteristics were comparable among the three groups, including age, sex, comorbidities, KDIGO risk category, serum albumin, proteinuria, kidney function, and baseline anti-PLA2R antibody status (all *p* > 0.05). The only significant difference was follow-up duration, which was shorter in treatment-naive patients than in the relapsing and treatment-refractory groups (median 17 vs. 41 vs. 35 months, respectively; *p* = 0.044).

RTX was generally well tolerated. During follow-up, infections occurred in 7 of 58 patients (12.1%), and only one patient required hospitalization because of infection. One patient experienced an infusion-related allergic reaction. No other serious adverse events were observed.

### 3.2. Clinical Response After Rituximab

**Figure 1:**
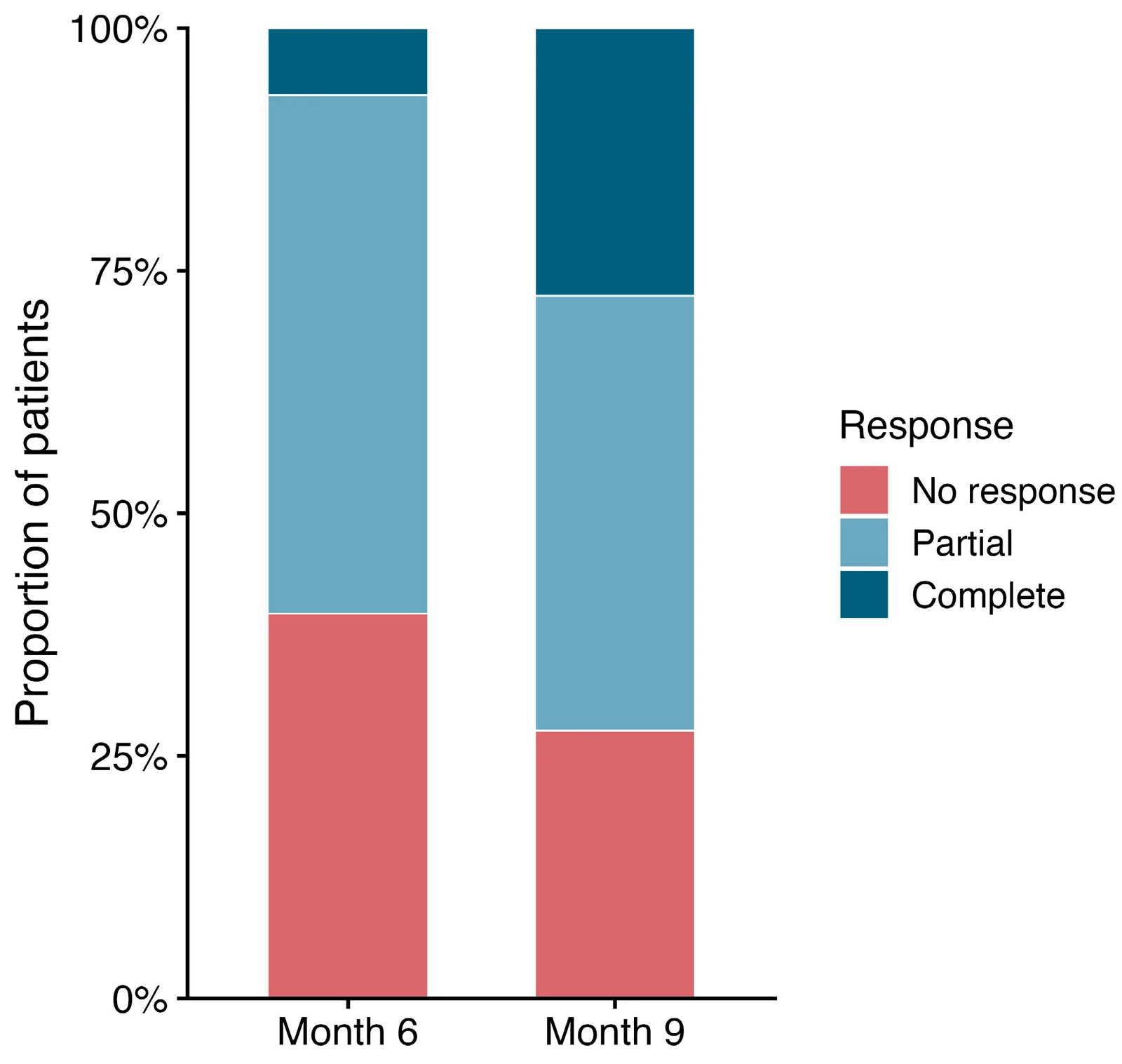
Changes in clinical response categories between the 6- and 9-month assessments following rituximab treatment. Stacked bars show the proportions of patients with non-response, partial remission, and complete remission at each time point.

Clinical response improved over time following RTX therapy (Figure 1). Overall remission rates increased from 60.3% at 6 months to 72.4% at 9 months (*p* < 0.001). Between the two assessments, 8 patients improved from non-response to partial remission and 13 from partial to complete remission, whereas only 1 patient worsened from partial remission to non-response.

**Figure 2:**
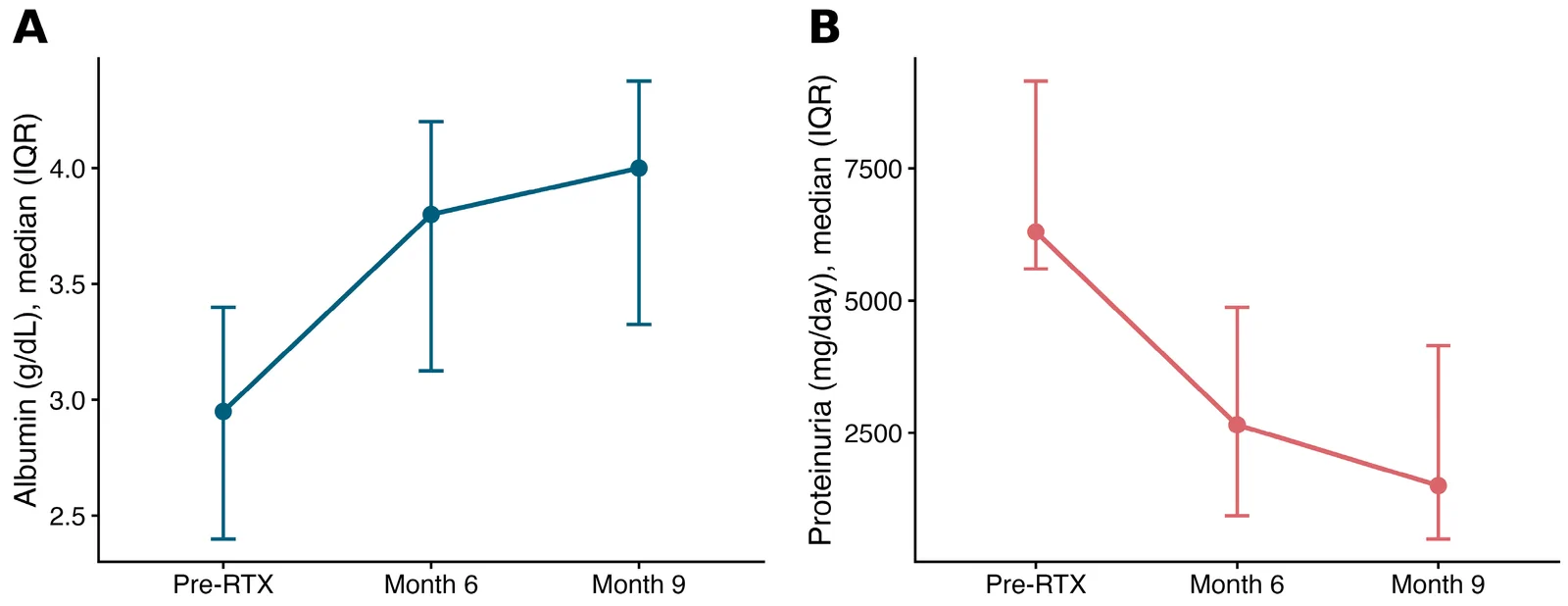
Longitudinal changes in serum albumin and proteinuria following rituximab treatment: (**A**) Median (interquartile range) serum albumin levels at baseline, 6 months, and 9 months after rituximab treatment. (**B**) Median (interquartile range) 24 h proteinuria at baseline, 6 months, and 9 months after rituximab treatment. RTX, rituximab.

Consistent with the improvement in clinical response, laboratory parameters also improved during follow-up (Figure 2). Median proteinuria decreased from 6300 (5600–9150) mg/day at baseline to 2650 (928–4875) mg/day at 6 months and further to 1500 (492–4150) mg/day at 9 months (*p* < 0.001). Serum albumin increased from 2.95 (2.40–3.40) g/dL at baseline to 3.80 (3.12–4.20) g/dL at 6 months and 4.00 (3.32–4.38) g/dL at 9 months (*p* < 0.001).

### 3.3. Anti-PLA2R Subgroup Analysis

Treatment outcomes were further analyzed according to baseline anti-PLA2R antibody levels (Table 2). Baseline anti-PLA2R antibody levels were significantly associated with clinical remission. Patients with anti-PLA2R antibody levels ≤ 150 RU/mL achieved significantly higher remission rates at both 6 and 9 months than those with levels > 150 RU/mL (*p* = 0.003 and *p* = 0.007, respectively). Although the overall treatment failure rate was comparable between the two groups, the pattern of treatment failure differed significantly (*p* = 0.015). Persistent treatment-refractory disease was more common in patients with anti-PLA2R antibody levels > 150 RU/mL, whereas relapse after remission occurred more frequently in those with anti-PLA2R antibody levels ≤ 150 RU/mL. Proteinuria reduction and serum albumin improvement were similar between the groups (Table 2).

### 3.4. Previous Treatment Status Subgroup Analysis

Treatment outcomes were also analyzed according to previous treatment status (Table 3). Notably, 6- and 9-month remission rates were comparable among treatment-naive, relapsing, and treatment-refractory patients. Likewise, complete remission at 9 months, proteinuria reduction, serum albumin improvement, and treatment failure rates did not differ significantly among the three groups (all *p* > 0.05). Consistent with these findings, the proportion of patients with persistent treatment-refractory disease was also similar across the three groups, occurring in 4 of 13 (30.8%) treatment-naive patients, 7 of 22 (31.8%) patients with relapsing disease, and 5 of 23 (21.7%) patients with treatment-refractory disease (*p* = 0.743).

RTX regimen characteristics also differed according to previous treatment status. Overall, 16 of 58 patients (27.6%) received RTX as monotherapy, while the remaining 42 (72.4%) received RTX in combination with corticosteroids and/or CNIs; the proportion receiving monotherapy did not differ significantly among treatment-naive (4/13, 30.8%), relapsing (9/22, 40.9%), and treatment-refractory (3/23, 13.0%) patients (Fisher’s exact *p* = 0.094). Concomitant corticosteroid use was similar across groups (8/13, 61.5%; 12/22, 54.5%; 12/23, 52.2%; *p* = 0.891), whereas concomitant CNI use differed significantly (3/13, 23.1%; 6/22, 27.3%; 15/23, 65.2%; *p* = 0.014), being more frequent among treatment-refractory patients. Additional RTX courses during follow-up were also more common among relapsing and treatment-refractory patients than treatment-naive patients (1/13, 7.7%; 10/22, 45.5%; 13/23, 56.5%; *p* = 0.012). Clinical outcomes did not differ significantly between patients who received RTX monotherapy and those who received combination therapy, including remission at 6 months (12/16, 75.0% vs. 23/42, 54.8%; *p* = 0.232), remission at 9 months (12/16, 75.0% vs. 30/42, 71.4%; *p* = 1.000), and treatment failure (8/16, 50.0% vs. 26/42, 61.9%; *p* = 0.552). These comparisons were exploratory, as treatment allocation was not randomized.

### 3.5. Predictors of Treatment Failure

Baseline characteristics were subsequently compared according to the pattern of treatment failure (Table 4). Patients with persistent treatment-refractory disease had higher baseline anti-PLA2R antibody levels, higher baseline proteinuria, and lower serum albumin levels than those who experienced relapse after remission (all *p* < 0.05). Age, KDIGO risk category, and previous treatment status were comparable between the groups.

Among the 16 patients with persistent treatment-refractory disease after RTX, 12 received cyclophosphamide during follow-up. Two had previously failed cyclophosphamide before RTX and remained refractory after RTX. Of the remaining 10 patients who received cyclophosphamide after RTX failure, 4 achieved partial remission, 2 achieved complete remission, and 4 remained treatment-refractory during follow-up.

**Figure 3:**
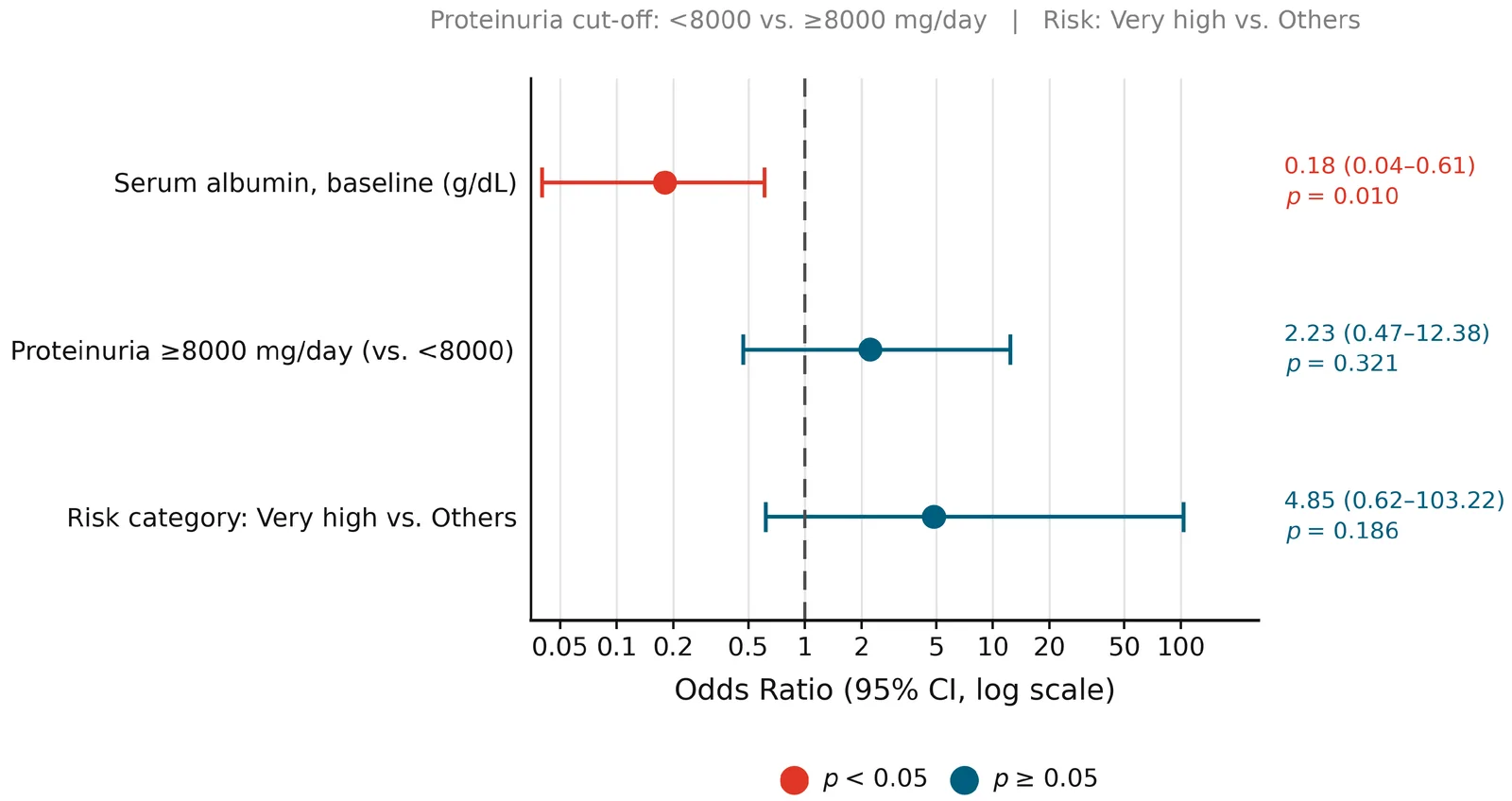
Multivariable logistic regression analysis of predictors of rituximab treatment failure. Forest plot depicting the multivariable-adjusted odds ratios (ORs) with 95% confidence intervals (CIs) for predictors of rituximab treatment failure. The *x*-axis is presented on a logarithmic scale, and the dashed vertical line represents OR = 1 (null effect). Red circles indicate statistically significant associations (*p* < 0.05); blue circles indicate non-significant associations. Proteinuria was dichotomized as ≥8000 mg/day vs. <8000 mg/day, and risk category was compared as very high vs. others.

Factors associated with treatment failure were assessed using logistic regression analysis (Table 5, Figure 3). In univariable analysis, lower baseline serum albumin and higher baseline proteinuria were significantly associated with treatment failure. Variables with a univariable *p*-value < 0.20, including the very high KDIGO risk category, were entered into the multivariable model. In multivariable analysis, only baseline serum albumin remained independently associated with treatment failure (OR 0.18, 95% CI 0.04–0.61; *p* = 0.010). Given the limited sample size, these multivariable findings should be considered exploratory.

The final multivariable model included three predictors (baseline albumin, proteinuria category, and risk category) relative to 34 treatment-failure events (24 non-events), corresponding to approximately 11.3 events per variable. Internal validation using bootstrap resampling (1000 resamples) yielded an apparent C-statistic of 0.802 and an optimism-corrected C-statistic of 0.775, indicating limited optimism, although the wide confidence interval obtained for the risk category variable reflects sparse-data instability that internal validation cannot correct.

### 3.6. Failure-Free Survival

**Figure 4:**
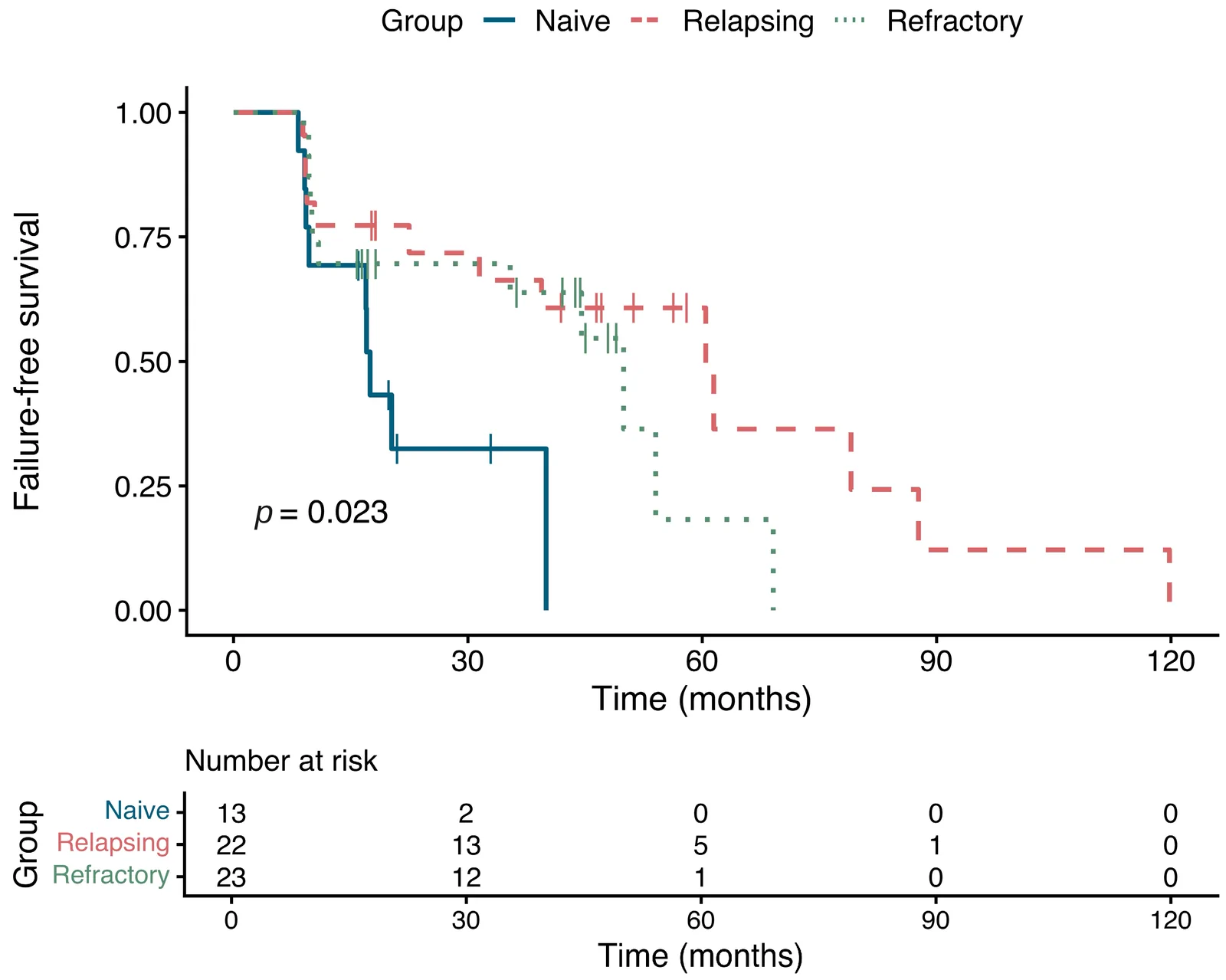
Kaplan–Meier analysis of failure-free survival according to previous treatment status. Tick marks indicate censored observations, and the numbers at risk for each group are shown below the plot. Groups were compared using the log-rank test. Line types (solid, dashed, and dotted) denote the naive, relapsing, and refractory groups, respectively, to aid group differentiation when the figure is printed or viewed in grayscale.

Failure-free survival according to previous treatment status was further evaluated using Kaplan–Meier analysis (Figure 4). A significant difference was observed among treatment-naive, relapsing, and treatment-refractory patients (log-rank *p* = 0.023).

To assess whether the observed difference persisted after adjustment for baseline serum albumin, an exploratory Cox proportional hazards model was fitted. After adjustment, the association between previous treatment status and failure-free survival was substantially attenuated and no longer statistically significant (relapsing vs. naive: adjusted HR 0.59, 95% CI 0.20–1.74, *p* = 0.341; refractory vs. naive: adjusted HR 0.51, 95% CI 0.20–1.26, *p* = 0.142), whereas lower baseline serum albumin remained independently associated with a higher risk of treatment failure (adjusted HR 0.20, 95% CI 0.10–0.42, *p* < 0.001). The proportional hazards assumption was not violated (Schoenfeld residuals, global *p* = 0.21). This is consistent with the absence of significant differences in remission and treatment-failure rates at the predefined 9-month assessment (Section 3.4). Given the limited sample size and the unequal follow-up across groups, this adjusted analysis should be considered exploratory.

## 4. Discussion

In this retrospective cohort study, RTX showed favorable clinical outcomes in patients with PMN. Clinical remission continued to improve between 6 and 9 months after treatment, consistent with the delayed therapeutic response to RTX. Lower baseline anti-PLA2R antibody levels were associated with higher remission rates, whereas baseline serum albumin was the only variable independently associated with treatment failure. Treatment outcomes were also comparable across treatment-naive, relapsing, and treatment-refractory patients.

One of the principal findings of our study was the continued improvement in clinical remission between 6 and 9 months after RTX treatment. This observation is consistent with the extended follow-up results of the GEMRITUX trial [3]. Similarly, the MENTOR trial demonstrated durable remission with RTX, supporting the role of RTX as a first-line treatment for PMN [4]. These findings suggest that assessing treatment response only at 6 months may underestimate the full therapeutic effect of RTX.

Another important finding of our study was the association between baseline anti-PLA2R antibody levels and clinical response to RTX. Patients with baseline anti-PLA2R antibody levels ≤ 150 RU/mL achieved significantly higher remission rates at both 6 and 9 months than those with higher antibody levels. Similar findings have been reported in real-world cohorts, where patients with baseline anti-PLA2R antibody levels > 150 RU/mL achieved lower remission rates following RTX therapy [7]. This threshold is also recognized as a marker of high immunological risk [8,9], consistent with previous studies demonstrating that higher baseline anti-PLA2R antibody titers are associated with a lower likelihood of remission after RTX treatment [10,11,12].

Interestingly, baseline anti-PLA2R antibody levels appeared to distinguish distinct patterns of treatment failure rather than the overall likelihood of treatment failure. Patients with higher antibody levels were more likely to remain treatment-refractory, whereas relapse after remission occurred more frequently in those with lower antibody levels. To our knowledge, this distinction has not been specifically evaluated in previous studies. Our findings extend the current understanding of anti-PLA2R as a prognostic biomarker and may inform post-treatment surveillance and individualized retreatment strategies, although prospective validation is required.

Baseline serum albumin was the only variable independently associated with treatment failure in our multivariable analysis. Our findings are consistent with a recent multicenter real-world study demonstrating that higher baseline serum albumin was associated with higher remission rates in patients with PMN [13]. Hypoalbuminemia reflects the severity of nephrotic syndrome and may better capture overall disease burden than proteinuria alone. This finding is biologically plausible because severe hypoalbuminemia has been associated with increased urinary loss of RTX, resulting in lower circulating drug concentrations and reduced treatment efficacy. Teisseyre et al. demonstrated that lower baseline serum albumin was associated with lower circulating RTX concentrations after treatment, which in turn were associated with a lower likelihood of achieving remission [14]. Together, these findings support the role of serum albumin as a practical predictor of treatment response in patients receiving RTX.

Our findings are in line with the post hoc analysis of the MENTOR trial reported by Barbour et al., in which the combination of anti-PLA2R antibody level and serum albumin measured after three months of treatment predicted treatment nonresponse better than antibody level alone or than assessment at baseline [15]. Our results point in the same direction at an earlier landmark: baseline anti-PLA2R antibody levels were associated with remission and with the pattern of treatment failure, whereas baseline serum albumin was the only independent predictor of failure, indicating that the two markers carry complementary rather than interchangeable prognostic information. Direct comparison is nonetheless limited, as that analysis included only anti-PLA2R–positive patients enrolled in a randomized trial and assessed the absence of remission at 12 months, whereas our real-world cohort also included anti-PLA2R–negative patients, and our primary outcome additionally encompassed relapse after remission. The 150 RU/mL threshold applied in the present study was adopted from previous reports rather than derived from our own data and therefore differs from the 323 RU/mL value identified in that analysis. Because follow-up antibody measurements were not routinely available, we could not reproduce this three-month reassessment; baseline antibody level and serum albumin may nonetheless provide prognostic information in settings where serial monitoring is not feasible.

Another notable finding was that RTX outcomes were comparable regardless of previous treatment status. Neither remission rates, treatment failure, nor improvements in laboratory parameters differed significantly among treatment-naive, relapsing, and treatment-refractory patients. These findings are consistent with the prospective matched-cohort study by Cravedi et al., who demonstrated similar reductions in proteinuria and comparable remission rates in patients receiving RTX as second-line therapy after failure or relapse following previous immunosuppression and in matched patients receiving first-line RTX [16]. Favorable outcomes of RTX have also been reported in patients with relapsed PMN [17]. In contrast, Zhang et al. reported higher remission rates in treatment-naive patients [7]. This discrepancy may be explained by the relatively small treatment-naive subgroup and its shorter follow-up period, largely reflecting changes in the national reimbursement policy for RTX in Türkiye. Although Kaplan–Meier analysis demonstrated differences in failure-free survival, remission rates and treatment failure at the predefined 9-month assessment were comparable, suggesting that differences in follow-up duration may have contributed to the observed survival differences. Overall, our findings support the use of RTX across different clinical presentations of PMN and suggest that previous treatment exposure alone should not discourage its use in appropriate patients.

In an exploratory multivariable Cox model adjusting for baseline serum albumin, the unadjusted difference in failure-free survival across treatment-history groups was no longer statistically significant, while lower baseline serum albumin remained independently associated with a higher risk of treatment failure. These findings suggest that the observed unadjusted survival difference may partly reflect differences in baseline disease severity and follow-up duration rather than an independent association with previous treatment status.

This study also has several limitations. First, its retrospective single-center design may have introduced selection bias, and the relatively small sample size limited the statistical power of subgroup analyses. Second, although baseline anti-PLA2R antibody measurement was available for all patients, serial measurements during follow-up were not, because testing was performed in private laboratories and required out-of-pocket payment. This precluded the assessment of immunological remission and of longitudinal changes in antibody levels. Prospective multicenter studies incorporating protocol-driven serial anti-PLA2R antibody measurements, together with the baseline variables identified in the present study, would allow a more dynamic and precise assessment of the response to RTX than any single baseline marker. Third, treatment response beyond 9 months was not included in the primary outcome analysis. Because of the retrospective design, many patients received additional RTX courses after the initial treatment according to clinical judgment, making later assessments less suitable for evaluating the response to the initial RTX regimen. Fourth, the treatment-naive group had a shorter follow-up period than the other groups. This difference largely reflected changes in the national reimbursement policy for RTX in Türkiye. Before 2023, RTX was mainly reserved for patients with relapsing or treatment-refractory disease after failure of first-line therapy, whereas reimbursement approval in 2022 enabled earlier use in treatment-naive patients. Consequently, comparisons involving the treatment-naive group, particularly time-to-event analyses, should be interpreted with caution. Fifth, the multivariable logistic regression model for treatment failure had a modest events-per-variable ratio (approximately 11.3), and although bootstrap internal validation indicated limited optimism (apparent C-statistic 0.802 vs. optimism-corrected 0.775), the possibility of residual overfitting cannot be fully excluded; the multivariable and adjusted survival findings should therefore be regarded as exploratory pending external validation. Sixth, combination therapy and retreatment were not randomly allocated but were determined by the treating physician, predominantly in patients with more severe disease or previous treatment failure. The comparison between RTX monotherapy and combination regimens is therefore susceptible to confounding by indication and should be regarded as descriptive. Finally, given the relatively small sample size and the number of secondary and subgroup comparisons, these findings should be considered exploratory and hypothesis-generating and require confirmation in larger, independent cohorts.

## 5. Conclusions

RTX achieved favorable clinical outcomes in patients with PMN, with continued improvement in remission beyond 6 months. Baseline anti-PLA2R antibody levels were associated with both remission and distinct patterns of treatment failure, whereas baseline serum albumin was the only variable independently associated with treatment failure in an exploratory multivariable model. These findings suggest that serum albumin and anti-PLA2R antibody levels provide complementary prognostic information that may improve risk stratification and individualized follow-up after RTX therapy. Prospective multicenter studies are warranted to validate these findings.

## Figures and Tables

**Table 1:** Baseline characteristics of the patients according to previous treatment status.

Variable	Overall Cohort (*n* = 58)	Treatment-Naive (*n* = 13)	Relapsing (*n* = 22)	Treatment-Refractory (*n* = 23)	*p*
Age, years	46 (36–53)	52 (44–63)	46 (39–53)	40 (27–52)	0.079
Male sex, *n* (%)	43/58 (74.1)	7/13 (53.8)	18/22 (81.8)	18/23 (78.3)	0.207
Diabetes mellitus, *n* (%)	7/58 (12.1)	2/13 (15.4)	1/22 (4.5)	4/23 (17.4)	0.451
Hypertension, *n* (%)	34/58 (58.6)	11/13 (84.6)	13/22 (59.1)	10/23 (43.5)	0.057
Serum albumin, g/dL	2.95 (2.40–3.40)	2.80 (2.30–3.00)	3.30 (2.55–3.58)	2.90 (2.25–3.10)	0.123
24 h proteinuria, mg/day	6300 (5600–9150)	6300 (6060–7290)	6190 (5275–9800)	6700 (5995–8800)	0.818
Serum creatinine, mg/dL	0.96 (0.81–1.30)	0.90 (0.73–1.68)	1.00 (0.85–1.38)	0.94 (0.84–1.08)	0.834
eGFR, mL/min/1.73 m^2^	90 (58–108)	78 (36–109)	88 (54–97)	92 (70–111)	0.365
Anti-PLA2R positivity, *n* (%)	47/58 (81.0)	9/13 (69.2)	19/22 (86.4)	19/23 (82.6)	0.482
Anti-PLA2R > 150 RU/mL, *n* (%)	26/58 (44.8)	6/13 (46.2)	8/22 (36.4)	12/23 (52.2)	0.624
Follow-up duration, months	21 (11–46)	17 (10–20)	41 (18–58)	35 (10–45)	0.044
KDIGO risk category, *n* (%)					
Moderate	12 (20.7)	4 (30.8)	5 (22.7)	3 (13.0)	0.679
High	38 (65.5)	7 (53.8)	15 (68.2)	16 (69.6)	0.679
Very high	8 (13.8)	2 (15.4)	2 (9.1)	4 (17.4)	0.679

Data are presented as median (interquartile range) or *n* (%). eGFR, estimated glomerular filtration rate; KDIGO, Kidney Disease: Improving Global Outcomes; PLA2R, phospholipase A2 receptor.

**Table 2:** Treatment outcomes according to baseline anti-PLA2R antibody levels.

Outcome	Anti-PLA2R ≤ 150 RU/mL (*n* = 32)	Anti-PLA2R > 150 RU/mL (*n* = 26)	*p*
Remission at 6 months	25/32 (78.1)	10/26 (38.5)	0.003
Remission at 9 months	28/32 (87.5)	14/26 (53.8)	0.007
Treatment failure	17/32 (53.1)	17/26 (65.4)	0.426
Pattern of treatment failure			
Persistent treatment-refractory disease	4/17 (23.5)	12/17 (70.6)	0.015
Relapse after remission	13/17 (76.5)	5/17 (29.4)	

Data are presented as *n* (%). Baseline anti-PLA2R measurements were available for all 58 patients; the ≤150 RU/mL group included patients with negative anti-PLA2R antibody results. Persistent non-response was assessed at 9 months, whereas relapse was assessed during subsequent follow-up after the achievement of remission. Percentages for the pattern of treatment failure are calculated among patients with treatment failure in each group. PLA2R, phospholipase A2 receptor.

**Table 3:** Rituximab treatment outcomes according to previous treatment status.

Outcome	Treatment-Naive (*n* = 13)	Relapsing (*n* = 22)	Treatment-Refractory (*n* = 23)	*p*
Remission at 6 months	8/13 (61.5)	15/22 (68.2)	12/23 (52.2)	0.580
Remission at 9 months	9/13 (69.2)	17/22 (77.3)	16/23 (69.6)	0.806
Complete remission at 9 months	3/13 (23.1)	9/22 (40.9)	4/23 (17.4)	0.217
Treatment failure	9/13 (69.2)	13/22 (59.1)	12/23 (52.2)	0.660
Persistent treatment-refractory disease	4/13 (30.8)	7/22 (31.8)	5/23 (21.7)	0.743

Data are presented as *n* (%).

**Table 4:** Baseline characteristics according to the pattern of treatment failure.

Variable	Persistent Treatment-Refractory (*n* = 16)	Relapse After Remission (*n* = 18)	*p*
Age, years	51 (34–60)	42 (27–53)	0.292
Baseline anti-PLA2R level, RU/mL	360 (156–570)	99 (58–202)	0.012
Baseline 24 h proteinuria, mg/day	8550 (7068–13,225)	6250 (5745–8075)	0.047
Baseline serum albumin, g/dL	2.30 (2.18–2.58)	2.95 (2.50–3.32)	0.010
KDIGO risk category (high + very high), *n* (%)	14/16 (87.5)	16/18 (88.9)	1.000
Previous treatment status, *n* (%)			
Treatment-naive	4 (25.0)	5 (27.8)	0.626
Relapsing	5 (31.2)	8 (44.4)	0.626
Treatment-refractory	7 (43.8)	5 (27.8)	0.626

Data are presented as median (interquartile range) or *n* (%). KDIGO, Kidney Disease: Improving Global Outcomes; PLA2R, phospholipase A2 receptor.

**Table 5:** Logistic regression analysis of factors associated with treatment failure.

Variable	Univariable			Multivariable		
	OR	95% CI	*p*	OR	95% CI	*p*
Age	0.99	0.95–1.03	0.652	—	—	—
Sex (female vs. male)	1.08	0.33–3.73	0.900	—	—	—
Serum albumin, baseline (g/dL)	0.14	0.04–0.41	0.001	0.18	0.04–0.61	0.010
Proteinuria ≥8000 mg/day (vs. <8000)	4.90	1.35–23.60	0.025	2.23	0.47–12.38	0.321
Serum creatinine, baseline (mg/dL)	1.64	0.60–5.78	0.379	—	—	—
eGFR, baseline (mL/min/1.73 m^2^)	1.00	0.99–1.02	0.800	—	—	—
Anti-PLA2R > 150 RU/mL	1.67	0.58–4.96	0.347	—	—	—
Risk category: very high vs. others	5.96	0.96–115.80	0.106	4.85	0.62–103.22	0.186

CI, confidence interval; eGFR, estimated glomerular filtration rate; OR, odds ratio; PLA2R, phospholipase A2 receptor. Proteinuria was categorized as <8000 mg/day vs. ≥8000 mg/day, and risk category was dichotomized as very high vs. others (moderate + high). Variables were selected for the multivariable analysis based on *p* < 0.20 in the univariable analysis; the KDIGO risk category was additionally retained a priori.

## Data Availability

The data presented in this study are not publicly available because they contain information that could compromise patient privacy and confidentiality. De-identified data are available upon request from the corresponding author, subject to appropriate institutional approval.

## Abbreviations

ACEi	angiotensin-converting enzyme inhibitor
anti-PLA2R	anti-phospholipase A2 receptor
ARB	angiotensin receptor blocker
CI	confidence interval
CKD-EPI	Chronic Kidney Disease Epidemiology Collaboration
CNI	calcineurin inhibitor
CR	complete remission
eGFR	estimated glomerular filtration rate
IQR	interquartile range
KDIGO	Kidney Disease: Improving Global Outcomes
OR	odds ratio
PMN	primary membranous nephropathy
PR	partial remission
RTX	rituximab
SGLT2i	sodium-glucose cotransporter-2 inhibitor
